# Microfluidics-enabled mesenchymal stem cell derived Neuron like cell membrane coated nanoparticles inhibit inflammation and apoptosis for Parkinson’s Disease

**DOI:** 10.1186/s12951-024-02587-1

**Published:** 2024-06-25

**Authors:** Tong Lei, Caifeng Li, Yang Liu, Zhao Cui, Shiwen Deng, Junxian Cao, Hongjun Yang, Peng Chen

**Affiliations:** 1 Department of Disease and Syndromes Research, Institute of Basic Theory for Chinese Medicine, China Academy of Chinese Medical Sciences, Beijing, Dongcheng District, 100700 China; 2 Beijing Key Laboratory of Traditional Chinese Medicine Basic Research on Prevention and Treatment for Major Diseases, Experimental Research Center, China Academy of Chinese Medical Sciences, Beijing, Dongcheng District, 100700 China; 3 Hunan Provincial Key Laboratory of Complex Effects Analysis for Chinese Patent Medicine, Yongzhou, Hunan Province 425199 China

**Keywords:** Microfluidic chip, Nanoparticle, Inflammations, Apoptosis, Parkinson’s disease

## Abstract

Parkinson’s disease (PD) is the second largest group of neurodegenerative diseases, and its existing drug treatments are not satisfactory. Natural cell membrane drugs are used for homologous targeting to enhance efficacy. In this study, microfluidic electroporation chip prepared mesenchymal stem cell-derived neuron-like cell membrane-coated curcumin PLGA nanoparticles (MM-Cur-NPs) was synthesized and explored therapeutic effect and mechanism in PD. MM-Cur-NPs can protect neuron from damage, restore mitochondrial membrane potential and reduce oxidative stress in vitro. In PD mice, it also can improve movement disorders and restore damaged TH neurons. MM-Cur-NPs was found to be distributed in the brain and metabolized with a delay within 24 h. After 1 h administration, MM-Cur-NPs were distributed in brain with a variety of neurotransmitters were significantly upregulated, such as dopamine. Differentially expressed genes of RNA-seq were enriched in the inflammation regulation, and it was found the up-expression of anti-inflammatory factors and inhibited pro-inflammatory factors in PD. Mechanically, MM-Cur-NPs can not only reduce neuronal apoptosis, inhibit the microglial marker IBA-1 and inflammation, but also upregulate expression of neuronal mitochondrial protein VDAC1 and restore mitochondrial membrane potential. This study proposes a therapeutic strategy provide neuroprotective effects through MM-Cur-NPs therapy for PD.

**Supplementary Information:**

The online version contains supplementary material available at 10.1186/s12951-024-02587-1.

## Graphical Abstract

Microfluidic chips prepared mesenchymal stem cell-derived neuron-like cell encapsulation promotes curcumin nanoparticles to improve mobility disorders in Parkinson’s disease.

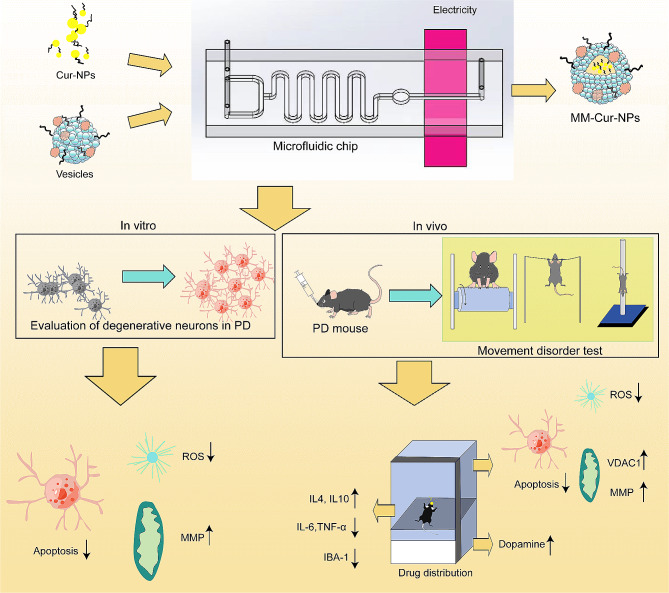

## Introduction

Parkinson’s disease (PD) is the second most common neurodegenerative disease after Alzheimer’s disease, and its common pathological features are the death of dopaminergic neurons in the substantia nigra and decreased dopamine secretion, resulting in symptoms such as tremors, limb stiffness, facial stiffness, and rigidity [1]. PD patients present with significant dopaminergic neuronal loss and decreased dopamine secretion, so almost treatment strategies revolve around dopamine callback [2]. Dopamine substitutes or receptor agonists are the most common PD drugs, but they briefly improve clinical motor symptoms rather than slowing or halting disease progression, and they have side effects such as hallucinations, cognitive impairment, memory loss, frontal lobe dysfunction, sleep disturbances, enteritis, and diarrhea [3]. The pathogenesis of PD is unclear and involves a variety of pathogenic factors such as oxidative stress, mitochondrial dysfunction, inflammation, autophagy, and toxicity of α-synuclein (α-Syn) aggregates [4]. Combination nanomedicines derived from natural biomaterials have a variety of advantages including bioavailability, targeting ability, bypassing the blood-brain barrier (BBB), improving oxidative stress and reducing inflammation, which have facilitated the research of novel therapies for PD [5].

Mesenchymal stem cells (MSCs), with high-speed proliferation, multi-lineage differentiation, homing and immunomodulation, are potential clinical materials for the treatment of PD [6]. MSCs, especially their natural cell membranes, which can be combined with biomaterials to construct drug delivery systems, have become a hotspot for disease treatment. We have studied the contents and neural differentiation of mesenchymal stem cells [7–9], but it is unclear whether the native cell membranes derived from them can be used as nanocarriers. The main advantages of MSCs and MSC-derived neuron-like cell membrane-encapsulated drugs are that the combination drugs can be specifically targeted and released to the disease site, and have good biosafety [10]. The coating of these MSC-derived cell membranes provides a novel drug treatment strategy for the treatment of diseases, and exerts multiple efficacy through synergistic drug functions [11, 12]. Curcumin (Cur) is a small natural molecule with a variety of activities, such as anti-inflammatory, neuroprotective and immunomodulating. Cur was found to be effective in promoting α-synuclein (α-Syn) clearance in 1-Methyl-4-phenyl-1,2,3.6-tetrahydropyridine (MPTP) induced PD mice [13, 14]. However, low solubility of Curcumin limits application, and enhancing solubility and targeting helps to improve the efficacy [15]. PLGA is a highly malleable nanomaterial that can be formulated into many types of nanomaterials. PLGA-coated nanoparticles can not only bypass the blood-brain barrier through size-based passive internalization osmosis to target drug delivery to the brain, but also improve drug targeting distribution to enhance efficacy [16, 17]. Microfluidic chips can help the high-speed preparation of nanomaterials due to their advantages of miniaturization, intelligence and integration [18]. In this study, we designed *m*icrofluidic electroporation chip prepared *m*esenchymal stem cell-derived neuron-like cell membrane-coated *cur*cumin PLGA *n*ano*p*article*s* (MM-Cur-NPs) to explore the synergistic efficacy of natural cell membrane-encapsulated drugs for PD.

## Materials and methods

### Reagents

Curcumin, PLGA (75: 25) purchased, polyvinyl alcohol (PVA) purchased from Macklin Company (Shanghai, China). 2’,7’-Dichlorodihydrofluorescein diacetate, (DCFH-DA) purchased from LABLEAD (Beijing, China). MPTP and 1-Methyl-4-phenylpyridinium iodide (MPP+) were obtained from Sigma-Aldrich (St. Louis, MO, USA). The apoptosis kit, mitochondrial membrane potential detection kit, autophagy kit, cell counting kit (CCK8), 4’,6-diamidino-2-phenylindole (DAPI) obtained from Beyotime Biotechnology (Shanghai, China). All antibodies including TH, IBA-1, TNF-α, VDAC1 and conjugated secondary antibodies were bought from Servicebio (Wuhan, China). Sulfo-Cyanine7 NHS (Cy7) were acquired from MeilunBio (Dalian, China).

### Microfluidic chip design and fabrication

All microfluidic chip channels were set to 1 mm in height and 1 mm in width. The circular incubator was 1 mm high and 4 mm in diameter. The aluminum electrode was placed in the electroporation area under the PC channel with a width of 10 mm and a thickness of 0.1 mm to ensure the electroporation effect of the fluid in the channel. Using standard soft lithography technology, a silicon wafer mold with designed pattern was made using photoresist. The aluminum electrode was fixed to the glass substrate by soft lithography and thermal evaporation. The PC layer was then cast from the silicon mold, punched holes, and finally bonded to an electrode-patterned glass substrate using an oxygen plasma system (Harrick Scientific, USA). All hot bonded form a closed flow channel. Finally, the device was heated at 120℃ for 2 days to ensure soundness and regain hydrophobicity.

### Preparation and characterization of MM-Cur-NPs

PLGA nanoparticles were prepared by water/oil/water-liquid solvent evaporation technology. PLGA was dissolved in acetone and then emulsified by phacoemulsification in 1% (w/v) PVA in water to produce a primary emulsion. The above emulsion then added to an aqueous solution containing 1% (w/v) PVA, and then sonicated again to form a secondary emulsion. Centrifuge and wash twice with deionized water. After drying, the nanoparticles were redispersed in acetone and slowly dripped into the curcumin solution. The nanoparticles were redispersed in 2 mL of deionized water by centrifugation at 15, 000 rpm, 20 min, 4℃ and washed twice with deionized water. Neuro-like cells were harvested with reference to our previous study [7]. Suspend cells in homogenization buffer containing Tris-HCl (pH = 7.5), KCl, sucrose, MgCl_2_, and protease/phosphatase inhibitors. The obtained mixture was crushed with a homogenizer IKA (T18) homogenizer. Centrifuge sequentially at 3, 000 g for 10 min and 10, 000 g sequentially at 4℃ for 30 min to obtain the suspension. The polyethylene tube was slowly introduced into the microfluidic chip by syringe pump (TS2 − 80, Longer Precision Pump, China) with 500 mg of PLGA nanoparticles and 1 mL of cell membrane for complete particle coating, respectively. The electrical pulse parameters refer to past reports and optimize the voltage and duration of the electrical pulse and the flow rate of the solution to achieve effective electroporation. When the duration and flow rate were 200 µs and 20 µL·min^− 1^, respectively, the voltage was regulated between 20 and 70 V. When the voltage and flow rate were 50 V and 20 µL·min^− 1^, respectively, the duration was adjusted from 50 µs to 300 µs. When the voltage and duration were 50 V and 200 µs, respectively, the flow rate was regulated in the range of 10–50 µL·min^− 1^. After electroporation, the resulting mixture was collected from the outlet and sonicated.

### Characterization of MM-Cur-NPs

The nanoparticle solution was droppedly onto the copper mesh, and after 30 min of sample deposition, rinse the grid twice with PBS. Add 5 µL of uranyl acetate stain to the nanoparticle-loaded grid. The morphology of these nanoparticles was characterized using transmission electron microscopy (TEM, JEOL, JEM-6700 F) applying an accelerating voltage of 120 KV. Dynamic light scattering (DLS) was used to determine the particle size of nanoparticles.

### Cell culture and viability

Human neuroblastoma cells SH-SY5Y, microglial cells BV2 were seeded in DMEM medium supplemented with 10% fetal bovine serum (FBS, BI, Israel), 1% 10,000 units/mL penicillin and 1% 10 mg/mL streptomycin (BI, Israel), and 100 µmol L-ascorbic acid (BI, Israel). Place the flask (Corning, USA) in a constant temperature incubator at 37℃ with 100% humidity and 5% CO_2_ concentration. Replace with fresh medium every 2–3 days and passage after a cell confluency of 80–90%. The in vitro models of PD constructed by pretreating SH-SY5Y cells and BV2 cells with MPP+ (4 mM) for 24 h, the effect of nanoparticles on cell viability was detected by CCK8 colorimetry. A total of 2 × 10^5^ cells/mL of SH-SY5Y cells and BV2 cells were seeded into 96-well plates, incubated at 37℃ for 1 h with CCK8 solution after 24 h of different drug treatment, and finally measured by microplate reader the absorbance generated at 450 nm. A total of 3 independent experimental replicates were performed.

### Detection of autophagy

SH-SY5Y and BV2 cells were seeded in 6-well plates, respectively, and after MPP + treatment for 24 h, different drugs were added for 24 h. Remove the medium, add MDC staining solution, incubate at 37 °C protected from light for 30 min in a cell culture incubator, wash 3 times with Assay Buffer, and measure the fluorescence intensity of each sample with fluorescence microscopy (EVOS, Thermofisher, USA).

### Reactive oxygen species analysis

SH-SY5Y and BV2 cells were seeded in 6-well plates, respectively, and after MPP + treatment for 24 h, different drugs were added for 24 h. Remove the medium, add 20 µM DCFH-DA staining solution and incubate at 37℃ for 30 min, wash 3 times with basal medium, and measure the fluorescence intensity of each group with fluorescence microscopy (EVOS, Thermofisher, USA).

### Mitochondrial membrane potential analysis

SH-SY5Y and BV2 cells were seeded in 6-well plates, respectively, and after MPP + treatment for 24 h, different drugs were added for 24 h. Remove the medium, add JC-1 staining solution and incubate at 37℃ for 30 min, wash 3 times with basal medium, and measure the mitochondrial membrane potential (MMP) fluorescence intensity of each group with fluorescence microscopy (EVOS, Thermofisher, USA).

### Detection of membrane proteins

Free vesicles or proteins were first removed from the sample solution by centrifugation and unbound proteins were removed by dialysis with a 30 nm porous membrane for 12 h. Proteins from mesenchymal stem cell-derived neuron-like cell membranes, cell membrane-coated nanoparticles, and individual nanoparticle groups were extracted through 95℃ for 5 min. After cooling at room temperature, 25 µL of sample from all groups was loaded into each well of the Bio-Rad electrophoresis system. Protein staining was done bright blue using Coomassie and destained with acetic acid overnight prior to imaging.

### Animals and treatment

The male C57BL/6 mice (6–8 weeks) were purchased from Charles River (Beijing, China). Animal experiments was carried out in accordance with the ARRIVE guidelines, and the China Academy of Chinese Medical Sciences Animal Care and Use Committee ethical standards and national guidelines. All procedures were approved by the Ethics Committee of China Academy of Chinese Medical Sciences for the use of experimental animals. All mice were maintained under static environment with a temperature of 22℃ ± 2℃, a humidity of 55-65%, and a light/dark cycle of 12 h, water and food were provided free of charge, and all animals were euthanized after the completion of the experiment. All mice were randomly divided into 5 groups (male, 6-8 weeks, *n* = 6), including control group, PD group, curcumin group, curcumin-PLGA nanoparticles group and MM-Cur-NPs group. MPTP-induced mouse models of PD were fabricated by daily intraperitoneal injection of MPTP (25 mg/kg/d) for 7 consecutive days. Group of Curcumin (Cur, 100 mg/kg/2d), curcumin-PLGA nanoparticles (Cur-NPs, 100 mg/kg/2d), MM-Cur-NPs (100 mg/kg/2d) were administered through the nasal cavity at a dose of for 4 weeks. Behavioral testing of all grouped mice was performed one week after the end of treatment.

### Behavioral analysis

All mice were performed to behavioral testing to assess the mobility function of mice. The following three behavioral experiments were tested:

Rotation rod test: Using the accelerated rotation device, train mice in accelerated mode (4-40 rpm) for three min continuously for three days before testing. Train at a constant rotational speed (16 rpm) until mice can stay on the rod for at least 100 s. Perform a formal experiment by placing mice on a rotating roller and speeding up from 4 rpm to 40 rpm in 3 min. The time of the first drop of the mouse is recorded to represent the length of time the mice stay on the rod. All mice were tested 3 times.

Pole test: A rough wooden pole with a diameter of 16 mm and a height of 60 cm was made, placed on the top of the mouse, and the time it took to climb to the bottom was recorded. All mice were kept for training starting 3 days prior to testing, and all mice were tested 3 times.

Suspension test: Place a thin horizontal line 40 cm from the table, pick up the mouse, and hang both front paws on the line. Two paws can be grasped as 3 points, single paws can be grasped as 2 points, and if they cannot be grasped, they were counted as 1 point. All mice were kept for training starting 3 days prior to testing, and all mice were tested 3 times at the time of formal trials.

### Distribution detection of nanoparticles

The fluorescent probe, Cy7, was used to evaluate the brain-targeted efficiency of MPTP to treat nanoparticles in PD mice. Three group Cy7, Cur-NPs, and MM-Cur-NPs were given nasally to assess the biodistribution of brain. Animals were imaged at 0, 1, 3, 6, 12, 24 h after administration in vivo, using Living Image software (Caliper, Alameda, CA).

### Detection of inflammatory factors

All mouse blood was allowed to stand and centrifuge (4℃, 3000 rpm, 20 min) to obtain serum. We refer to the manufacturer’s protocol of ELISA kits from Jiangsu Meimian Industrial Co., Ltd (Jiangsu, China). All serum levels of IL-4, IL-10, IL-6, TNF-α were measured.

### Histological analysis

Histochemical analysis was performed 5 weeks after the start of the first nanoparticle treatment. For histopathological analysis, tissue samples (lung, liver, kidney, spleen, heart, and brain) were collected immediately after euthanasia in all mice. Samples were routinely fixed in 10% buffered formalin, embedded in paraffin, and sectioned approximately 5 μm. Lung, liver, kidney, spleen, heart were used to stain with common hematoxylin and eosin (H&E). Microscopic study of tissue sections by light microscopy (Olympus-CH30, Japan) to identify possible histopathological lesions. All mouse brain tissue was sliced into a coronal plane. Striatum and substantia nigra regions were determined by serial slices with reference to brain region coordinates. Midbrain tissue was used to detect TH in the substantia nigra by conjugating secondary antibody with HRP and developing color by DAB. The striatum of the midbrain was individually embedded for the detection of TH, IBA-1, TNF-α, and VDAC1 by conjugated secondary antibodies with fluorescent dyes. The high-resolution immunofluores cence results were scanned and saved using a confocal microscope (Leica Biosystems). The intensity of the fluorescence was detected in the chemiluminescence analyzer and analyzed with ImageJ software.

### Microdialysis analysis

On the third day after modeling, the PD mice were anesthetized by inhalation of 2.0% isoflurane (flow rate 0.5 L·min^− 1^), and under the guidance of stereotaxic instrument, probe cannula was embedded in mice striatum (2.2 mm before the front halogen, 1.5 mm next to the middle seam, 2.25 mm into the needle) and fixed with dental cement. Place mice in a freely mobile device (33 cm× 40 cm× 36 cm) and perfuse 2 probe pathways with modified Ringer solution overnight at a flow rate of 0.3 µL·min^− 1^. The next day, adjust the flow rate to 1.3 µL·min^− 1^, equilibrate for 1.5 h, and start collecting the dialysate. MM-Cur-NPs were then administered nasally and the dialysate was collected continuously at the same volume and frequency. Collect 1 tube every 20 min with a collection volume of 26 µL. The control group was PD mice. High performance liquid-fluorescence chromatography was used to detect metabolite content in mouse brain dialysate to observe the effect of drugs on neurotransmitters in dialysate (Table S1).

### RNA sequencing

Midbrain tissue samples were obtained from mice, RNA was extracted by the Trizol method, and RNA quality was detected using NanoDrop2000. Enrich eukaryotes’ mRNA with magnetic beads containing Oligo(dT), followed by the addition of fragmentation buffe to randomly interrupt the mRNA. Using mRNA as a template, the first strand of cDNA was synthesized with random primers. Buffer, dNTPs, and DNA polymerase I were then added to synthesize the second strand of cDNA. Double-stranded cDNA was purified using AMPure XP beads. The purified double-stranded cDNA was then end-repaired, A-tailed, and ligated sequencing linkers, followed by fragment size selection with AMPure XP beads. Finally, PCR enrichment was performed to obtain the final cDNA library. The library was tested for quality, and machine sequencing was carried out only after the test results meet the requirements. The raw image data files obtained by high-throughput sequencing were converted into the original sequencing sequence by CASAVA Base Calling Sequenced Reads. Results were stored in the FASTQ file format, which contains sequence information for sequencing sequences (reads) and their corresponding sequencing quality information. Finally, HISAT2 software was used to sequence compare Clean Reads with the specified genome to obtain its position information on the reference genome.

### Bioinformatics analysis

Use featureCounts software to calculate the FPKM value expressed in each sample for each factor. DESeq2 was used for differential expression analysis of genes, and the *p*-value obtained from the original hypothesis test was corrected. The default was to use padj < 0.05, |log2(fold change)|>1 as the criteria for screening differentially expressed genes. Gene ontology (GO) enrichment and pathway analysis were used to understand the physiological properties of this protein using Database for Annotation, Visualization and Integrated Discovery (https://david.ncifcrf.gov/tools.jsp), including cellular components (CC), molecular functions (MF), biological processes (BP). The Kyoto Encyclopedia of Genes and Genomes (KEGG) analysis was used to annotate gene pathways.

### Statistical analysis

The data was mainly analyzed using GraphPad Prim 9.0 software. The t-test was used for comparisons between the two groups, and one-way ANOVA was used for three or more groups. *: *p* < 0.05; **: *p* < 0.01; ***: *p* < 0.001; ns, no significant.

## Results and discussion

### Synthesis and characterization of nanoparticles

**Fig. 1:**
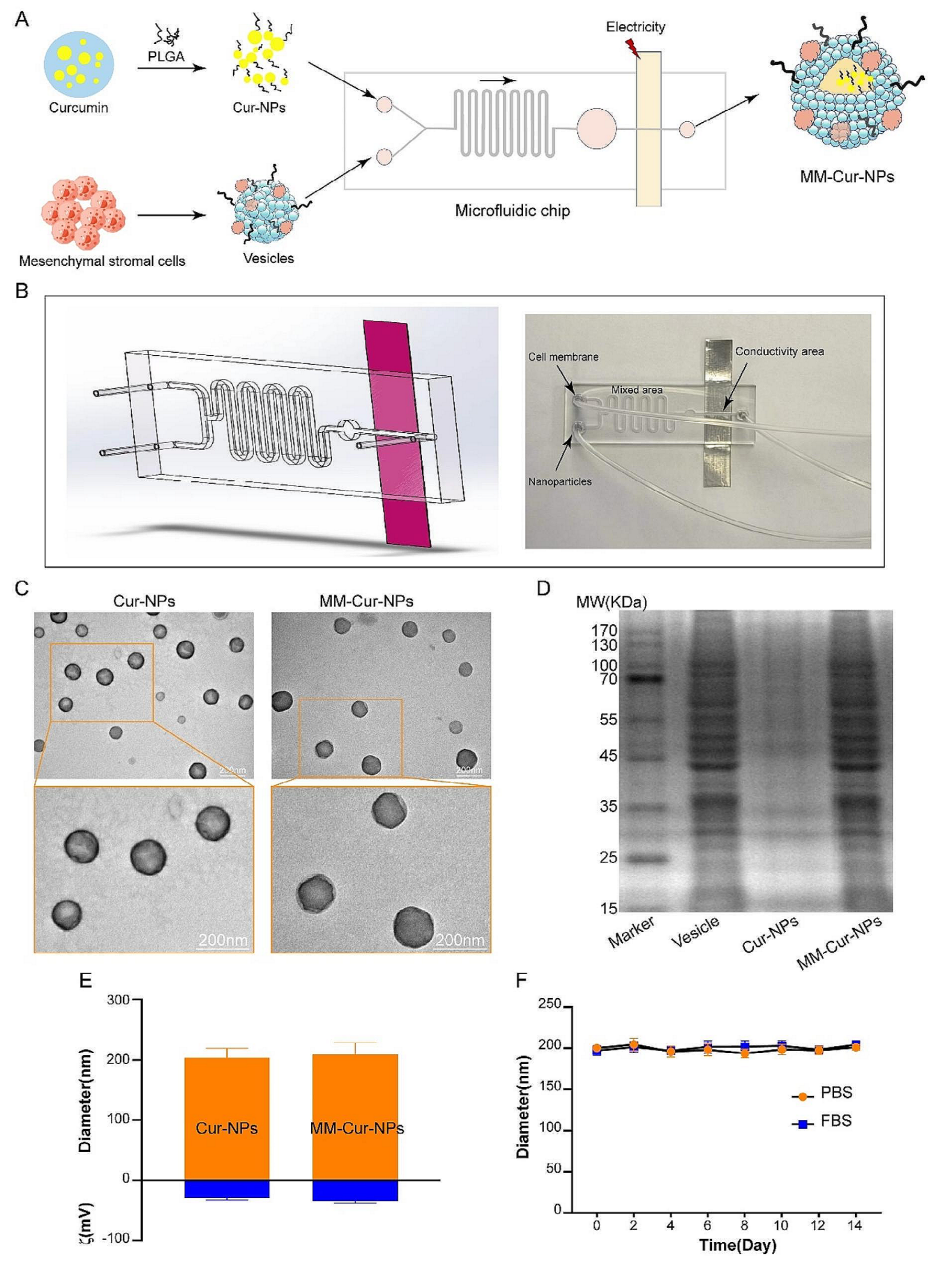
Preparation and characterization of nanoparticles. (**A**) Schematic of the preparation process of curcumin nanoparticles coated with mesenchymal stem cell-derived neuron-like cell membrane derived by microfluidic electroporation. (**B**) Design and preparation of microfluidic chips. (**C**) Transmission electron microscopy image of nanoparticles. Scale bars = 200 nm. (**D**) SDS-PAGE protein analysis of mesenchymal stem cell-derived neuron-like cell membrane vesicles, Cur-NPs and MM-Cur-NPs. (**E**) Size (diameter) and surface zeta potential (ζ) of Cur-NPs and MM-Cur-NPs. (**F**) Stability of MM-Cur-NPs in PBS and FBS by measuring the particle size. *n* = 3. The data are mean ± SD

We first prepared mesenchymal stem cell-derived neuron-like cell membrane-coated curcumin PLGA nanoparticles by microfluidic electroporation chip. As shown in the flowchart (Fig. 1A), after encapsulated by PLGA, curcumin was perfused into a channel of the microfluidic chip, and another channel flows into the extracted mesenchymal stem cell-derived neuron-like cell membrane. The microfluidic chip was developed and prepared for electroporation, which is mainly composed of five main parts including two inlets, a Y-shaped merging channel, an a S-shaped mixing channel, an a electroporation zone, and an outlet (Fig. 1B). The two samples converged through the microfluidic chip channel, flow together through the meandering incubation cell, where they were mixed twice, and finally the drug was wrapped through the conductive zone, and the final sample was collected. The conductive region of microfluidic chip was connected with cyclic electric pulse to enhance the efficiency of drug coating by slow-flowing electroporation [19]. The extraction of mesenchymal stem cell-derived neuron-like cell membranes was referred to the previous report [20]. Briefly, neurons are lysed with a hypotonic solution and centrifuged to obtain a cell membrane. The TEM image shown in Fig. 1C showed the appearance of the nanoparticles, and the MM-Cur-NPs were spherical nucleo-shell structures of about 200 nm, reflecting that the curcumin nanoparticle nuclei were encapsulated in a thin shell of the cell membrane.

Subsequently, sodium dodecyl sulfate polyacrylamide gel electrophoresis (SDS-PAGE) was used to assess the protein content of MM-Cur-NPs with the neuron-like cell membrane and bare curcumin pellets as background controls (Fig. S1). The results of the protein separation plot showed that most of the composition of the membrane protein was retained during the whole particle synthesis process, indicating that the protein profile on the surface of curcumin nanoparticles was successfully modulated (Fig. 1D). In addition, DLS measurements showed that Cur-NPs were 203.6 ± 15.4 nm in diameter and MM-Cur-NPs were 209.2 ± 18.8 nm in diameter after being coated with the cell membrane (Fig. 1E). The increase in the size of Cur-NPs corresponds to a cell membrane of 5–10 nm, indicating the successful coating of the neuron-like cell membrane [21]. Moreover, after coating the cell membrane, the zeta potential (ζ) of curcumin nanoparticles changed from 29.25 ± 3.30 mV to 33 ± 2.58 mV. Neuron-like cell vesicles-encapsulated nanoparticles exhibit higher absolute zeta potential compared to curcumin nanoparticles [11, 22]. MM-Cur-NPs was separately dispersed in phosphate-buffered saline (PBS) and fetal bovine serum (FBS) for two weeks to observe its long-term stability, and it was monitored for changes in particle size with DLS. As shown in Fig. 1F, the change in size does not significantly indicate the stability of MM-Cur-NPs. On overall, these measurements demonstrate the successful overlay of mesenchymal stem cell-derived neuron-like cell membranes on curcumin nanoparticles with long-term stability.

### The efficacy and intracerebral distribution of MM-Cur-NPs for PD mice

**Fig. 2:**
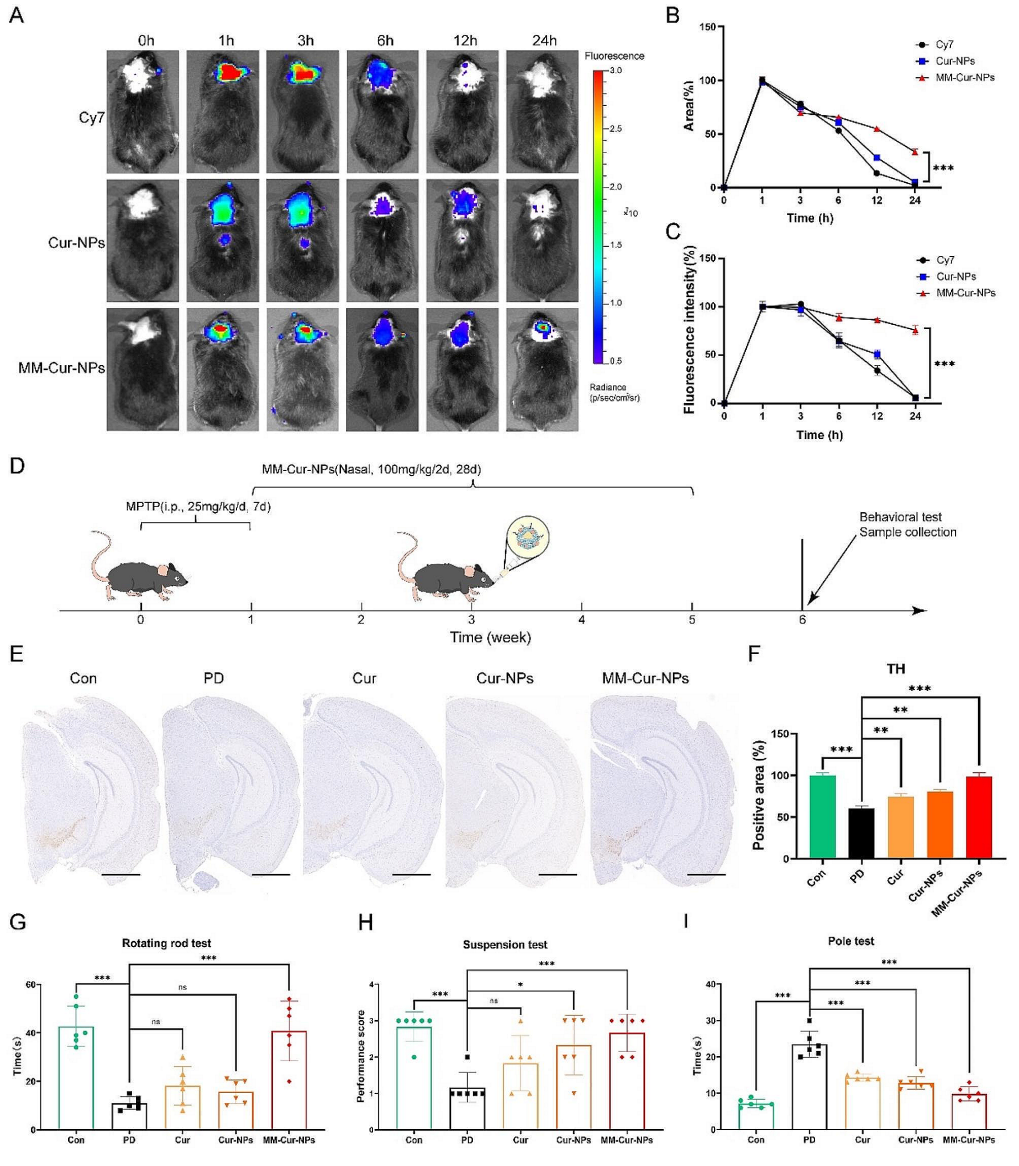
Distribution and efficacy evaluation of nanomaterials in MPTP-induced PD mice. (**A**) Distribution of nanoparticles within the brain of PD mice within 24 h after nasal administration (*n* = 6). (**B**) Statistical analysis of the positive area distribution of nanoparticles in the brain of PD mice. (**C**) Fluorescence intensity statistics of nanoparticle distribution in PD mouse brain. (**D**) Time flow chart of nanoparticle drug treatment in PD mice. (**E**) TH immunohistochemical staining of the substantia nigra region of mouse brain. Scale bars = 1000 μm. (**F**) TH-positive areas in the substantia nigra compact. (**G**) Rotation rod test of mice treated with different nanoparticles. The ordinate is the time to complete the test. (**H**) Suspension test of mice. The ordinate is the score of the mice. (**I**) Pole test of all mice. The ordinate is the time to complete the test. *n* = 6. The data are mean ± SD; *P < 0.05; **P < 0.01; ***P < 0.001 compared to the control group

MSCs have hypoimmunogenicity and the ability to target lesion sites, which may provided from cell membranes. The MSC-derived neuron-like cell membranes coating can retain most of the molecular repertoire expressed on the surface of the original neuron-like cells, retaining these intrinsic targeting capabilities and promoting drug accumulation at the lesion site [23, 24]. The MSC-derived neuron-like cell membranes can promote the retention of nanoparticles in vivo and prolong their circulating half-life. MSC-derived neuron-like cell membranes-encapsulated nanomaterials can bypass the BBB by expressing adhesion molecules, such as VCAM-1, and are able to target sites of inflammation [25, 26]. We examined whether MM-Cur-NPs has the ability to bypass the BBB and detect its distribution in brain tissue. The direct therapeutic effect of nanomaterials on brain regions may provide the basis for treatment. We found that the nanomaterials could enter the brain region. The fluorescence area and fluorescence intensity of MM-Cur-NPs reached their maximum at 1 h, and the cell membrane encapsulation prolonged the residence time compared with the nanoparticle group (Fig. 2A-C). In vitro fluorescence detection showed that Cy5.5-labeled MM-Cur-NPs could be taken up by cells and observed in mouse brain sections after nasal administration (Fig. S2-3). The cell membrane coating confers the nanoparticles with the ability to bypass the BBB [24]. Subsequently, we underwent MPTP for 7 consecutive days and then gave nanodrugs for one month (Fig. 2D). The PD patients are characterized by loss of dopaminergic neurons in the substriatum nigra and striatum, and tyrosine hydroxylase (TH) was a rate-limiting enzyme for dopamine synthesis. The results showed that MPTP treatment significantly damaged TH + neurons in the substantia nigra dense part of PD mice, but significantly recovered TH neuronal damage by MM-Cur-NPs (Fig. 2E-F). Additionally, the results of behavioral analysis showed that compared with healthy mice, PD mice showed a significant reduction in Rotation rod test time, a significant decrease in suspension score, and a significant extension of pole time. MM-Cur-NPs treatment significantly improved Rotation rod test time, suspension score and pole time for PD mice (Fig. 2G-I). Furthermore, with the development of nanoparticles in the treatment of biological systems, toxicological evaluation is emphasized to ensure their safety at the nanoscale [27]. These data suggest that MM-Cur-NPs can be targeted into and distributed in the brain, reducing TH nerves in the substantia nigra and improving behavioral performance with security in PD mice.

**Fig. 3:**
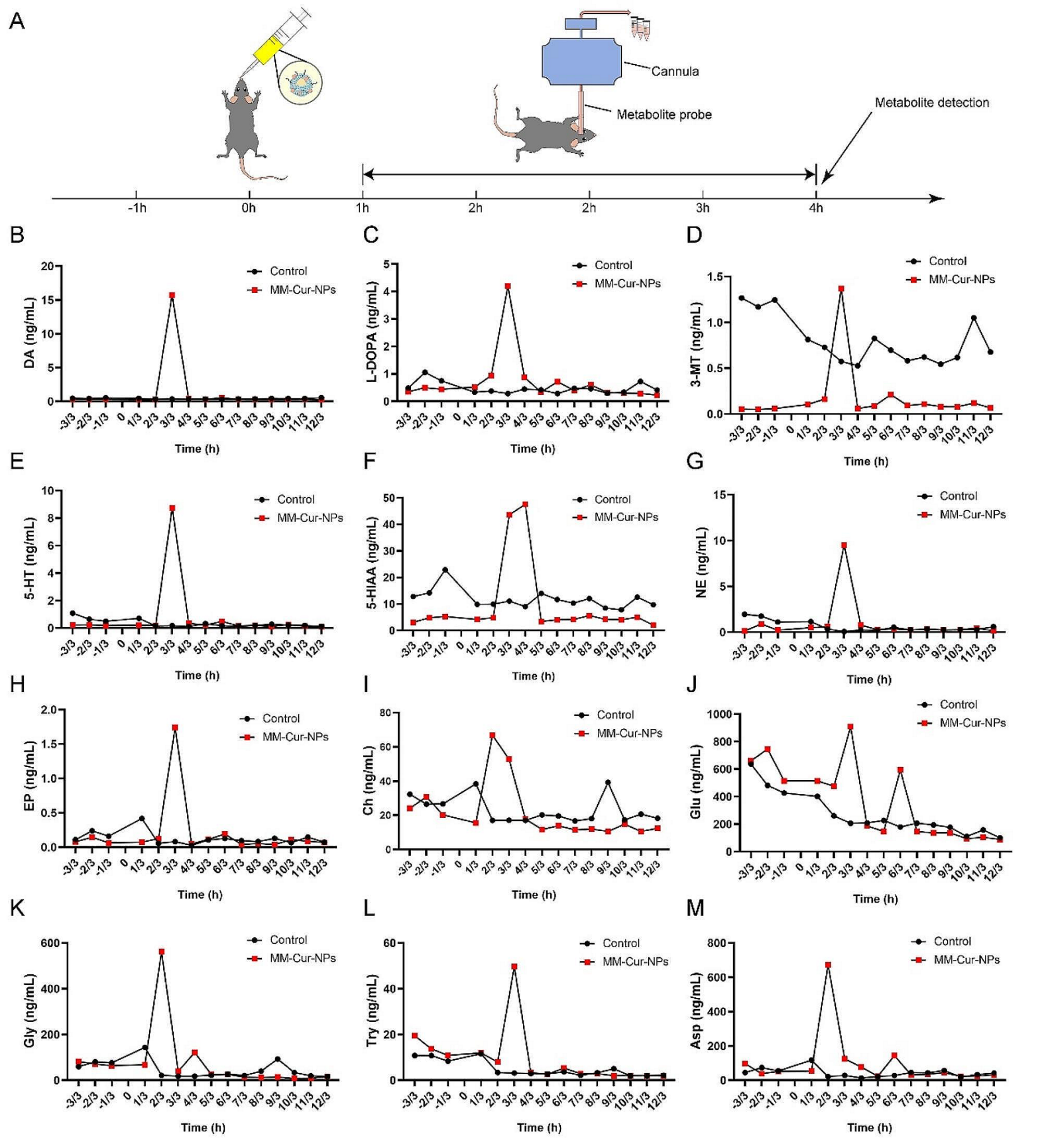
Metabolite and mRNA analysis of the effects of MM-Cur-NPs on brain tissue in PD. (**A**) Flow chart of microdialysis analysis. (**B-M**) Microdialysis analysis of the effect of MM-Cur-NPs on neurotransmitter metabolites in brain tissue in PD within 4 h

### MM-Cur-NPs promotes the recovery of dopamine secretion in the striatum of PD mice

Decreased DA secretion due to dopaminergic neuronal damage in the substantia nigra striatum region is a classic characterization of PD patients. MPTP, as a potent neurotoxin, induces preferential loss of ventral and lateral ganglion neurons in the substantia nigra region in PD mice [4]. MPTP-induced severe loss of dopaminergic neurons with a significant reduction in the average amount of DA in the striatum [28]. Microdialysis is an advanced real-time monitoring technique for active mice in the brain, which is used to detect dopamine secretion in PD mice. Pre-experiments on dopamine secretion found that dopamine levels fluctuated rapidly and remained stable within 4 h (data not shown). So we collected changes in PD metabolites within 4 h of dosing treatment and analyzed changes in a variety of neurotransmitter metabolites (Table S1). The results showed significant upregulation of dopamine, accompanied by fluctuations in multiple neurotransmitters (Fig. 3A). Three tubes of microdialysis samples were collected each hour and neurotransmitter concentrations were analyzed 1 h before dosing and 4 h post-dosing treatment in PD mice. The results showed that metabolites showed obvious large oscillations, including DA, L-DOPA, 3-MT, 5-HT, 5-HIAA, NE, EP, CH, GLU, GLY, TRY, and ASP. Almost all metabolites appear significantly upregulated 1 h after administration (Fig. 3B-M). The fluctuations in these values matched the observations of the intracerebral distribution of the nanomaterials, suggesting that MM-Cur-NPs treatment entered the BBB and affected dopamine secretion in the nigrastriatum.

### MM-Cur-NPs affected the inflammatory response in PD mice

**Fig. 4:**
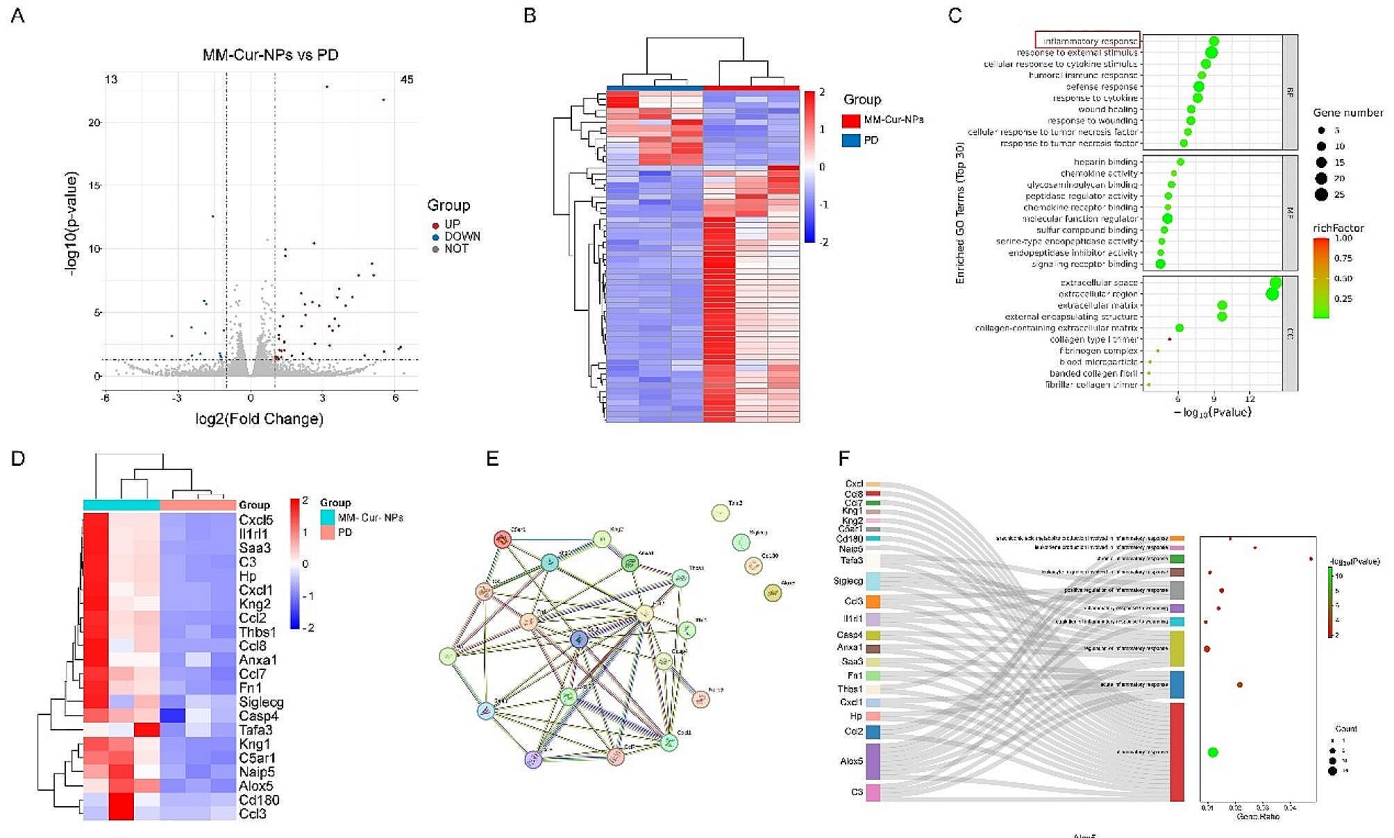
mRNA analysis of the effects of MM-Cur-NPs on midbrain tissue in PD. (**A**) Differential expression by transcriptomics to calculate mRNA expression by MM-Cur-NPs to brain tissue in PD. (**B**) Cluster heat map of differentially expressed genes. (**C**) GO analysis of differentially expressed genes. (**D**) Cluster heat map of differentially upregulated expressed genes associated with inflammation in the MM-Cur-NPs treatment group and PD group. (**E**) Interaction network analysis of differentially upregulated expressed genes associated with inflammation. (**F**) Differentially upregulated expressed genes associated with inflammation and their associated inflammatory pathways are analyzed in biological function analysis of GO assays

We next explored the effect of nanoparticles on molecular expression of PD through transcriptomics and we analyzed mRNA expression of midbrain tissue in PD mice after the treatment of MM-Cur-NPs. We found that a total of 35,277 genes were identified, and the results of differential expression gene (DEGs) calculation showed that compared with the PD group, MM-Cur-NPs treatment upregulated 45 genes and downregulated 17 genes (Table. S2). The clustering heatmap shows the within-group consistency of DEGs and the significance of differences between groups (Fig. 4A-B). We performed GO analysis to understand the genetic function, localization, and biological function of differential DEGs. A strong association of all DEGs in inflammation was found, including extracellular space (*p* = 3.76409E-14), extracellular region (*p* = 6.70922E-14), chemokine activity (*p* = 3.91422E-12), immune response (*p* = 8.31152E-12), cell chemotaxis (*p* = 1.44776E-11), inflammatory response (*p* = 1.73338E-11), response to chemokine (*p* = 1.89217E-11), cellular response to chemokine (*p* = 1.89217E-11), defense response (*p* = 2.55106E-11) and granulocyte chemotaxis (*p* = 5.96745E-11) (Fig. 4C). The inflammatory response was highlighted in the GO analysis results. So we extracted all genes involved in inflammation regulation in upregulated DEG, and clustered the results to find that a large number of inflammatory responses were upregulated and had a complex network of social regulation (Fig. 4D-E). GO analysis showed that these inflammatory regulatory pathways mainly included inflammatory response, acute inflammatory response, regulation of inflammatory response, regulation of inflammatory response to wounding, inflammatory response to wounding, positive regulation of inflammatory response, leukocyte migration involved in inflammatory response, chronic inflammatory response, leukotriene production involved in inflammatory response, and arachidonic acid metabolite production involved in inflammatory response (Fig. 4F). Besides, KEGG pathway analysis found that DEG involved a large number of regulatory-level pathways. We extracted the signaling pathways that up-regulated DEG and found that the improvement in PD symptoms associated with MM-Cur-NPs treatment may benefit from the regulation of multiple signaling pathways, including IL-17 signaling pathways, Chemokine signaling pathway, PI3K-Akt signaling pathway, TNF signaling pathway, Inflammatory mediator regulation of TRP channels, Neuroactive ligand-receptor interaction, cGMP-PKG signaling pathway, p53 signaling pathway, TGF-beta signaling pathway, and NF-kappa B signaling pathway (Fig. S4-5). These results suggest that MM-Cur-NPs may improve symptoms in PD mice by upregulating a series of inflammation-responsive molecules and signaling pathways in brain tissue.

**Fig. 5:**
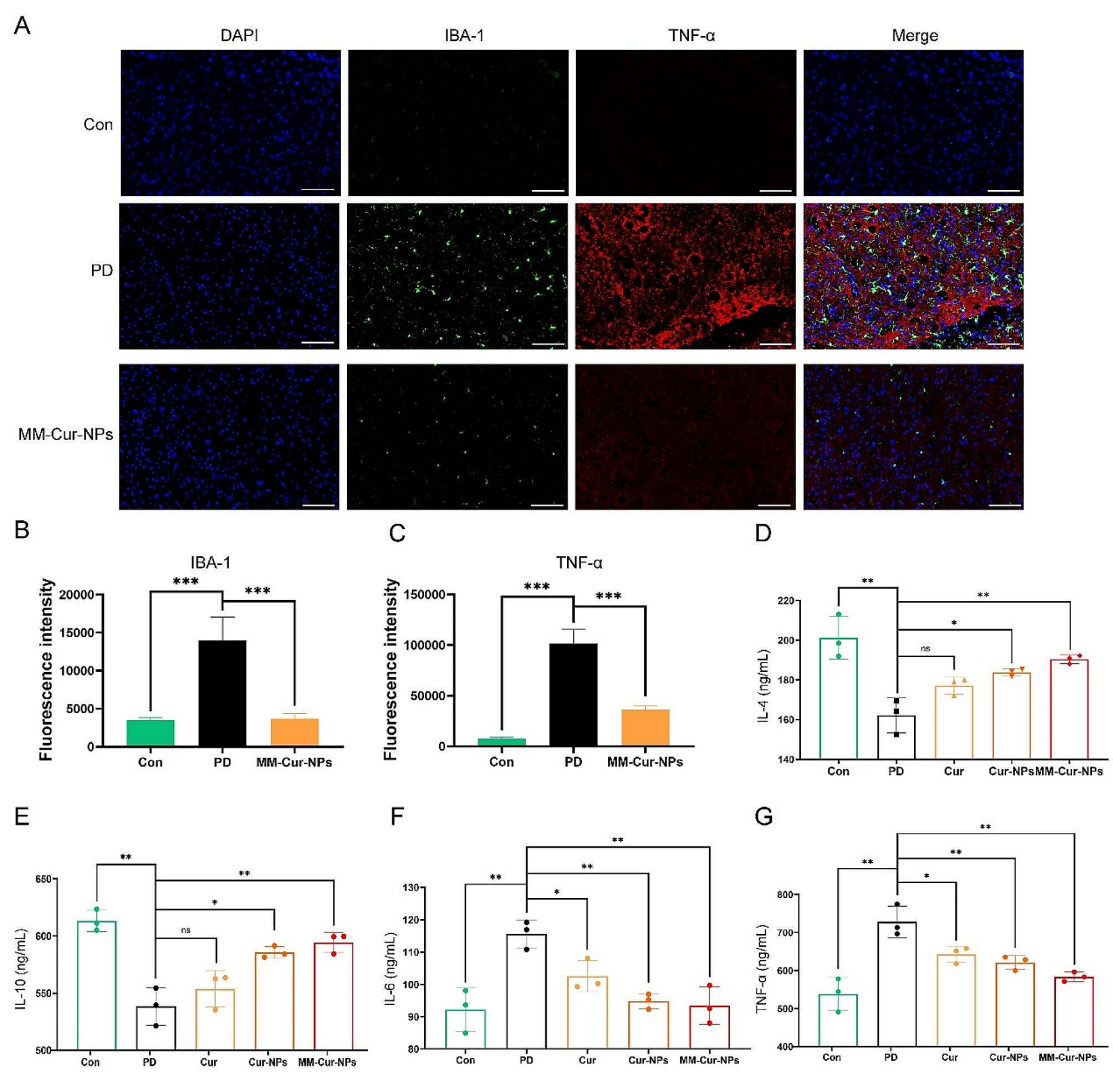
Effect of MM-Cur-NPs on inflammation in the brain and peripheral inflammation. (**A**) Immunofluorescence analysis of MM-Cur-NPs for striatal IBA-1 and TNF-α expression in PD mice. Scale bars: 100 μm. (**B**) Statistical analysis of positive expression of IBA-1. (**C**) Statistical analysis of positive expression of TNF-α. (**D**) Serum IL-4 expression in PD mice. (**E**) Serum IL-10 expression in PD mice. (**F**) Serum IL-6 expression in PD mice. (**G**) Serum TNF-α expression in PD mice. Con: control group; PD: PD group; Cur: curcumin group; Cur-NPs: curcumin-PLGA nanoparticles group; MM-Cur-NPs: MM-Cur-NPs group. *n* = 6. The data are mean ± SD; *P < 0.05; **P < 0.01; ***P < 0.001 compared to the control group

### MM-Cur-NPs inhibited inflammation in PD mice

Transcriptomics suggested the potential function of inflammation in mediating the efficacy of MM-Cur-NPs. Due to the association of microglia with inflammation [29], we first examined the effect of MM-Cur-NPs on microglia. The results showed microglia hyperplasia in PD mice (Fig. 5A), which was similar to what had been previously reported [30]. The results showed that upregulation of the microglial marker IBA-1 and TNF-α expression were inhibited after MM-Cur-NPs treatment (Fig. 5B-C). Inflammation are associated with Reactive oxygen species (ROS) and are also regulated by mitochondria [31, 32]. 1-methyl-4-phenyl-1,2,5,6-tetrahydropyridine (MPTP), a neurotoxin, was accidentally discovered to cause function in humans with subacute parkinsonism [33]. It can bypass the blood-brain barrier. It can be absorbed by cells, such as neurons, and be metabolized into the toxic metabolite MPP + to inhibit the activity of mitochondrial complex I, leading to an increase in oxygen radicals and dopaminergic neuronal death [34]. MPP + and MPTP has been applied to the preparation of a variety of in vitro and in vivo PD models [35, 36]. MPP + was used for PD molding in 4 mM for 24 h, and the results showed that MPP+ (PD group) resulted in significant death in half of the human neuroblastoma SH-SY5Y and mouse microglial BV2 cell lines. We next measured ROS and mitochondrial membrane potential (MMP) in the glial cell line, BV2. The results showed that MPP + promoted ROS accumulation and mitochondrial damage in BV2 cells, which is a classic PD cell model. After MM-Cur-NPs treatment, it was found that ROS was inhibited and MMP was repaired in BV2 (Fig. S6). In addition, we examined the expression of inflammatory factors in the serum of PD mice. The results showed that MM-Cur-NPs inhibited the upregulation of pro-inflammatory factors IL6 and TNF-α in PD mice, and upregulated the anti-inflammatory factors IL4 and IL10 (Fig. 5D-G). These results suggest that MM-Cur-NPs can inhibit microglial proliferation and inhibit systemic inflammation in PD mice, and these benefits may be brought by curcumin [37].

### MM-Cur-NPs promotes neuronal mitochondrial repair and reduces apoptosis

**Fig. 6:**
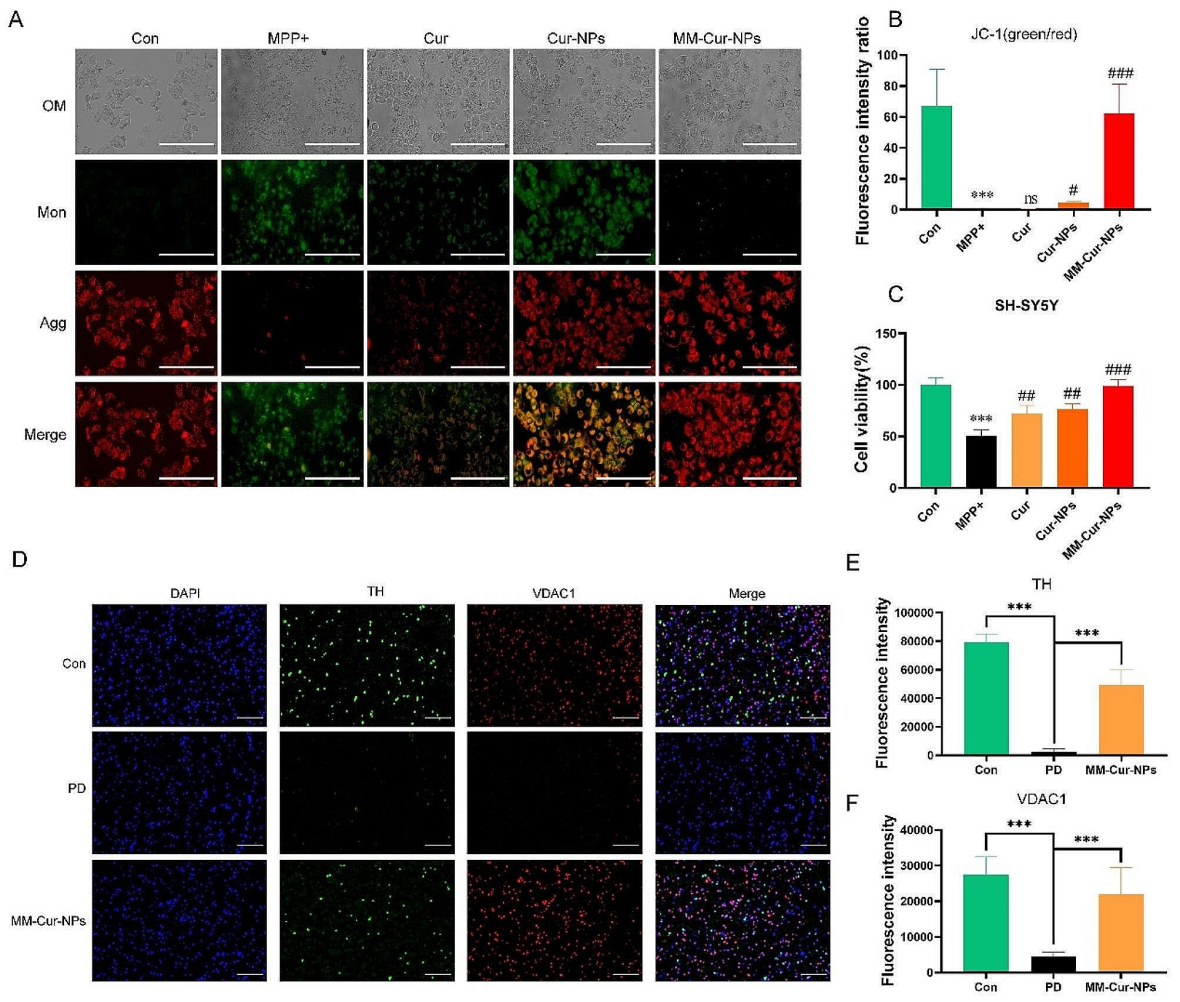
Effects of MM-Cur-NPs on the mitochondria of neurons. (**A**) Mitochondrial staining observation of SH-SY5Y by JC-1 staining. Scale bars: 200 μm. (**B**) Statistical plot of the ratio of green fluorescence and red fluorescence of JC-1 for SH-SY5Y. (**C**) Cell viability assay of SH-SY5Y cells by MPP + treatment and different nanoparticle treatments. The data are mean ± SD; *P < 0.05; **P < 0.01; ***P < 0.001 compared to the control group. #*P* < 0.05; # #*P* < 0.01; # # #*P* < 0.001 compared to the MPP + group. (**D**) Immunofluorescence analysis of the effects of MM-Cur-NPs on TH positive cells and mitochondrial markers in the striatum of PD mice. Scale bars: 100 μm. (**E**) Statistical analysis of the TH. (**F**) Statistical analysis of the mitochondrial marker VDAC1. The data are mean ± SD; *P < 0.05; **P < 0.01; ***P < 0.001

Neuronal mitochondrial injury is closely related to the pathogenesis of PD, which regulates a variety of pathological processes in PD, especially inflammation and ROS [38]. It was found that MM-Cur-NPs exhibited mitochondrial damage of PD in vitro (Fig. 6A-B). Treatment of MPP + leads to damage to neuronal cells, SH-SY5Y, and MM-Cur-NPs provides neuronal protection in PD cell models (Fig. 6C). TUNEL results showed that MPTP caused massive apoptosis for PD mice, but MM-Cur-NPs treatment inhibited apoptosis in the striatal region and substantia nigra (Fig. S7-8). The immunofluorescence results showed that the inhibition of the mitochondrial marker VDAC1 were upregulated in the striatum of PD mice (Fig. 6D-F). After treatment with MM-Cur-NPs, the expression of VDAC1 was restored. In PD cell models, we also found that MM-Cur-NPs reduced ROS accumulation (Fig. S9). These results suggest that MM-Cur-NPs ameliorate mitochondrial damage in TH-positive neurons and reduce neuronal apoptosis.

## Conclusion

We synthesized a natural cell membrane-coated nanoparticle, MM-Cur-NPs, to investigate the synergistic efficacy in PD models. Result found that it can protect SH-SY5Y and BV2 cells from MPP + damage, restore mitochondrial membrane potential and reduce oxidative stress in vitro. In PD mice, the coverage of the MSC-derived neuron-like cell membranes facilitates the distribution and accumulation of nanoparticles in brain, restoring damaged TH neurons and improving movement disorders. It also cause fluctuations of a variety of neurotransmitters metabolites. Transcriptomics results of midbrain tissue found that it can upregulated 45 genes and downregulated 17 genes, which regulate inflammatory response. An it promoted the expression of anti-inflammatory factors and inhibited pro-inflammatory factors. Immunofluorescence found that MM-Cur-NPs can not only reduce neuronal apoptosis, inhibit the microglial marker IBA-1 and inflammation, but also upregulate expression of neuronal mitochondrial protein VDAC1 and restore mitochondrial membrane potential. In summary, this study developed a synergistic nanoparticle, MM-Cur-NPs, to provides neuronal protective function and improves movement disorders for PD.

### Electronic supplementary material

Below is the link to the electronic supplementary material.

Supplementary Material 1

## Data Availability

The data that support the findings of this study are available from the corresponding author upon reasonable request.
