# Supplementary material for: Microfluidics-enabled mesenchymal stem cell derived Neuron like cell membrane coated nanoparticles inhibit inflammation and apoptosis for Parkinson’s Disease
Source: J Nanobiotechnology. 2024 Jun 25;22:370. doi: 10.1186/s12951-024-02587-1 (PMC11197265; doi:10.1186/s12951-024-02587-1)
Supplement: Supplementary file 1 — Supplementary Material 1 [file 12951_2024_2587_MOESM1_ESM.docx]

**Supporting Information**

**Microfluidics-enabled Mesenchymal Stem Cell Derived Neuron Like Cell Membrane Coated Nanoparticles Inhibit Inflammation And Apoptosis For Parkinson's Disease**

**Tong Lei^1*^, Caifeng Li^2^, Yang Liu^2^, Zhao Cui^2^, Shiwen Deng^2^, Junxian Cao^2^, Hongjun Yang^2,3*^, Peng Chen^2,3*^**

**Supplementary material**

Supplement Table 1(Table S1). Name and detailed description of the metabolite.

| **ID** | **Name** | **HMDB** | **PubChem** | **Annotation** |
| --- | --- | --- | --- | --- |
| DA | Dopamine | HMDB0000101 | 13730 | Neurotransmitter |
| L-DOPA | 3,4-Dihydroxyphenylalanine | HMDB0000181 | 6047 | DA precursor |
| 3-MT | 3-Methoxytyramine | HMDB0000022 | 1669 | DA metabolite |
| 5-HT | Serotonin | HMDB0000259 | 5202 | Neurotransmitter |
| 5-HIAA | 5-Hydroxyindoleacetic acid | HMDB0000763 | 1826 | 5HT metabolite |
| NE | Norepinephrine | HMDB0000216 | 439260 | Neurotransmitter; DA metabolite |
| EP | Epinephrine | HMDB0000068 | 5816 | Neurotransmitter; NE metabolite |
| Ch | Choline | HMDB0000097 | 305 | Precursor to ACh |
| Glu | Glutamate | HMDB0000148 | 33032 | Neuroactive amino acid |
| Gly | Glycine | HMDB0000123 | 750 | Neuroactive amino acid |
| Asp | Aspartate | HMDB0000191 | 5960 | Neuroactive amino acid |
| Try | Tryptophan | HMDB0000929 | 6305 | 5HT precursor; Kyn precursor |

Table S2. Differentially expressed genes in MM-Cur-NPs compared to PD.

| gene_id | symbol | log2FoldChange | pvalue |
| --- | --- | --- | --- |
| ENSMUSG00000017002 | Slpi | 3.1705 | 5.055E-28 |
| ENSMUSG00000040026 | Saa3 | 5.535 | 1.0921E-26 |
| ENSMUSG00000083061 | Gm12191 | -1.5626 | 2.5784E-17 |
| ENSMUSG00000024529 | Lox | 2.6519 | 5.4222E-15 |
| ENSMUSG00000001506 | Col1a1 | 1.4676 | 2.0113E-14 |
| ENSMUSG00000023885 | Thbs2 | 1.4438 | 7.8524E-14 |
| ENSMUSG00000035385 | Ccl2 | 5.0552 | 3.3593E-13 |
| ENSMUSG00000095180 | Rhox5 | 5.1255 | 3.596E-12 |
| ENSMUSG00000024678 | Ms4a4d | 4.4955 | 3.529E-12 |
| ENSMUSG00000031722 | Hp | 3.692 | 6.0112E-11 |
| ENSMUSG00000002020 | Ltbp2 | 2.1112 | 1.6157E-10 |
| ENSMUSG00000029371 | Cxcl5 | 4.24 | 3.4109E-10 |
| ENSMUSG00000024164 | C3 | 3.6155 | 3.8466E-10 |
| ENSMUSG00000083411 | Rpl30-ps10 | -1.9298 | 8.1732E-10 |
| ENSMUSG00000060143 | Gm10076 | 2.5728 | 1.188E-09 |
| ENSMUSG00000023043 | Krt18 | -1.8334 | 1.7866E-09 |
| ENSMUSG00000029816 | Gpnmb | 2.2573 | 2.0988E-09 |
| ENSMUSG00000031740 | Mmp2 | 2.8586 | 3.0294E-09 |
| ENSMUSG00000060459 | Kng2 | 3.9575 | 3.0164E-09 |
| ENSMUSG00000066407 | Gm10263 | 2.2935 | 1.8691E-08 |
| ENSMUSG00000040152 | Thbs1 | 1.3505 | 2.3272E-08 |
| ENSMUSG00000029380 | Cxcl1 | 3.5039 | 4.4361E-08 |
| ENSMUSG00000026069 | Il1rl1 | 3.6683 | 1.9038E-07 |
| ENSMUSG00000072940 | Gm10443 | 1.2301 | 2.1141E-07 |
| ENSMUSG00000099980 | Gm5619 | 3.2617 | 2.1561E-07 |
| ENSMUSG00000032363 | Adamts7 | 2.1165 | 2.1884E-07 |
| ENSMUSG00000017607 | Tns4 | -2.4555 | 2.7849E-07 |
| ENSMUSG00000044254 | Pcsk9 | -1.1111 | 4.8349E-07 |
| ENSMUSG00000035373 | Ccl7 | 3.4157 | 5.663E-07 |
| ENSMUSG00000017723 | Wfdc2 | -1.8826 | 9.196E-07 |
| ENSMUSG00000055172 | C1ra | 1.1808 | 1.4713E-06 |
| ENSMUSG00000020911 | Krt19 | -3.2738 | 0.000001942 |
| ENSMUSG00000032334 | Loxl1 | 1.1842 | 3.8542E-06 |
| ENSMUSG00000033581 | Igf2bp2 | 1.4225 | 7.3067E-06 |
| ENSMUSG00000026193 | Fn1 | 1.4039 | 8.9857E-06 |
| ENSMUSG00000096842 | Gm10736 | 2.6689 | 0.000013231 |
| ENSMUSG00000039209 | Rpl39l | 3.2916 | 0.000020634 |
| ENSMUSG00000101502 | Rpl7a-ps10 | 6.2468 | 0.000033856 |
| ENSMUSG00000073177 | Gm773 | 6.1739 | 0.000050466 |
| ENSMUSG00000109233 | Gm44866 | 1.1986 | 0.000066934 |
| ENSMUSG00000029661 | Col1a2 | 1.419 | 0.000068911 |
| ENSMUSG00000059775 | Rps26-ps1 | 1.2871 | 0.000084578 |
| ENSMUSG00000022875 | Kng1 | 5.5451 | 0.000098915 |
| ENSMUSG00000101431 | Gm7901 | -1.271 | 0.0001629 |
| ENSMUSG00000012428 | Steap4 | 2.1894 | 0.00017626 |
| ENSMUSG00000021697 | Depdc1b | -2.0886 | 0.00019197 |
| ENSMUSG00000074280 | Gm6166 | 4.7545 | 0.00028182 |
| ENSMUSG00000049382 | Krt8 | -2.439 | 0.00028366 |
| ENSMUSG00000097068 | Gm26760 | 1.7136 | 0.00028924 |
| ENSMUSG00000082809 | Gm14150 | -1.261 | 0.00029269 |
| ENSMUSG00000104037 | Gm37776 | 1.0397 | 0.00038641 |
| ENSMUSG00000020592 | Sdc1 | -1.0156 | 0.00041688 |
| ENSMUSG00000114277 | Gm48583 | -1.2174 | 0.00047517 |
| ENSMUSG00000109404 | Gm19963 | 1.2855 | 0.00048773 |
| ENSMUSG00000107620 | Gm44256 | 1.1245 | 0.00049252 |
| ENSMUSG00000038521 | C1s1 | 2.4735 | 0.000748 |
| ENSMUSG00000030074 | Gxylt2 | 1.0614 | 0.00077332 |
| ENSMUSG00000027868 | Tbx15 | 1.1854 | 0.00088715 |


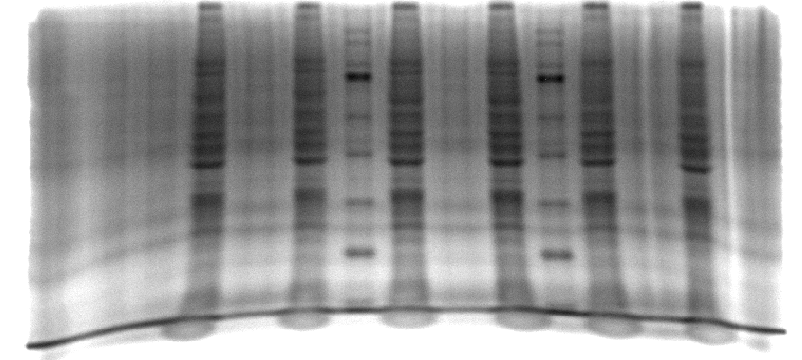


Fig S1. Uncropped gel for Figure 1. A total of 11 lanes were sampled. From left to right are mesenchymal stem cell membrane, Cur-NPs and MM-Cur-NPs, Protein marker, mesenchymal stem cell membrane, Cur-NPs and MM-Cur-NPs.


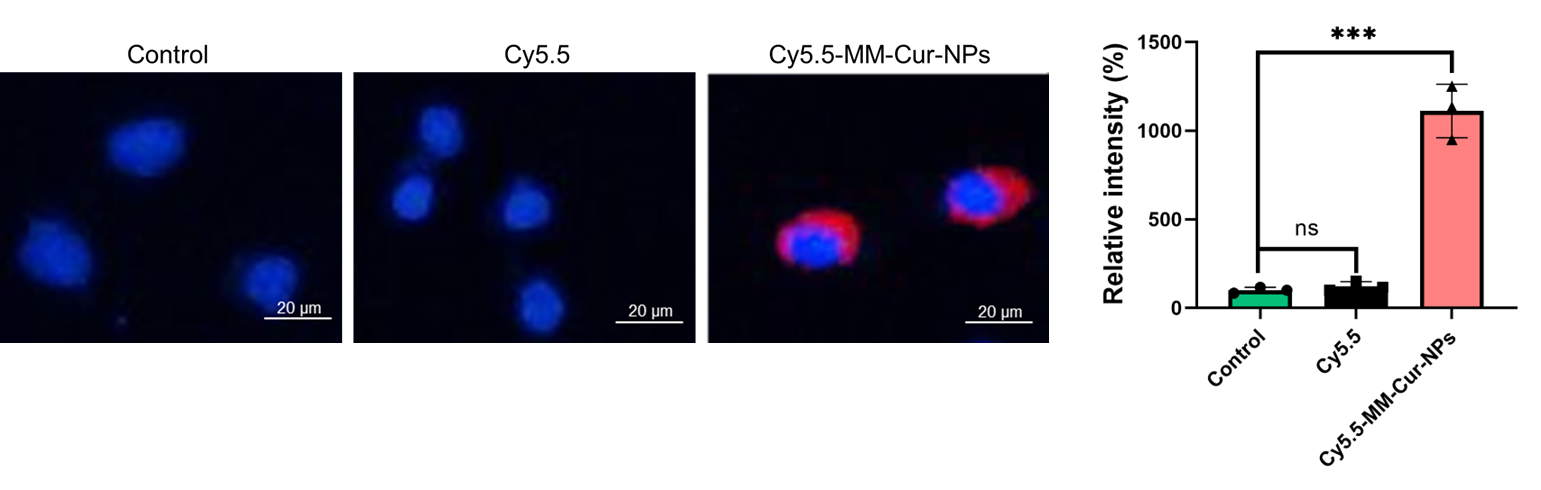


Fig S2. Representative fluorescence image of the SH-SY5Y cell model. MM-Cur-NPs were labeled with Cy5.5 (Red), and Neuronal nuclei were stained with DAPI (Blue). Scale bar: 20 μm. Quantification of fluorescence intensity of Cy5.5 (right). n = 3. The data are mean ± SD; **P* < 0.05; ***P* < 0.01; ****P* < 0.001.


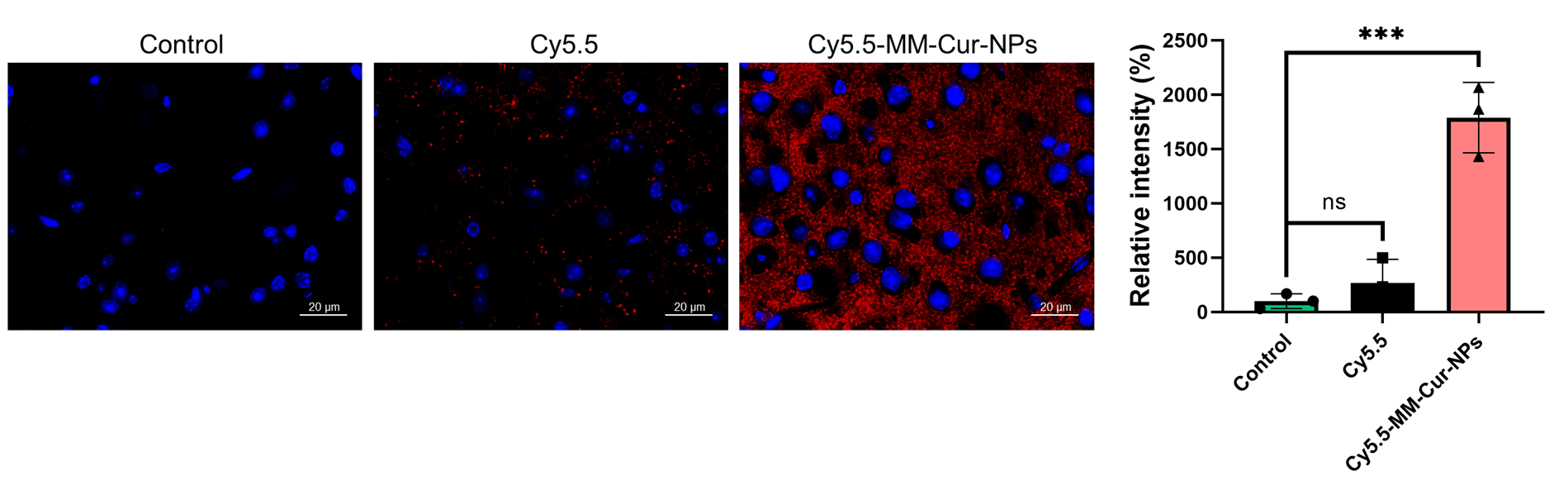


Fig S3. Fluorescence images of the PD mouse, 6 h after Nasal administration of Cy5.5-labeled MM-Cur-NPs (Red); Neuronal nuclei were stained with DAPI (Blue). Scale bar: 20 μm. Quantification of fluorescence intensity of Cy5.5 (right). n = 3. The data are mean ± SD; **P* < 0.05; ***P* < 0.01; ****P* < 0.001.


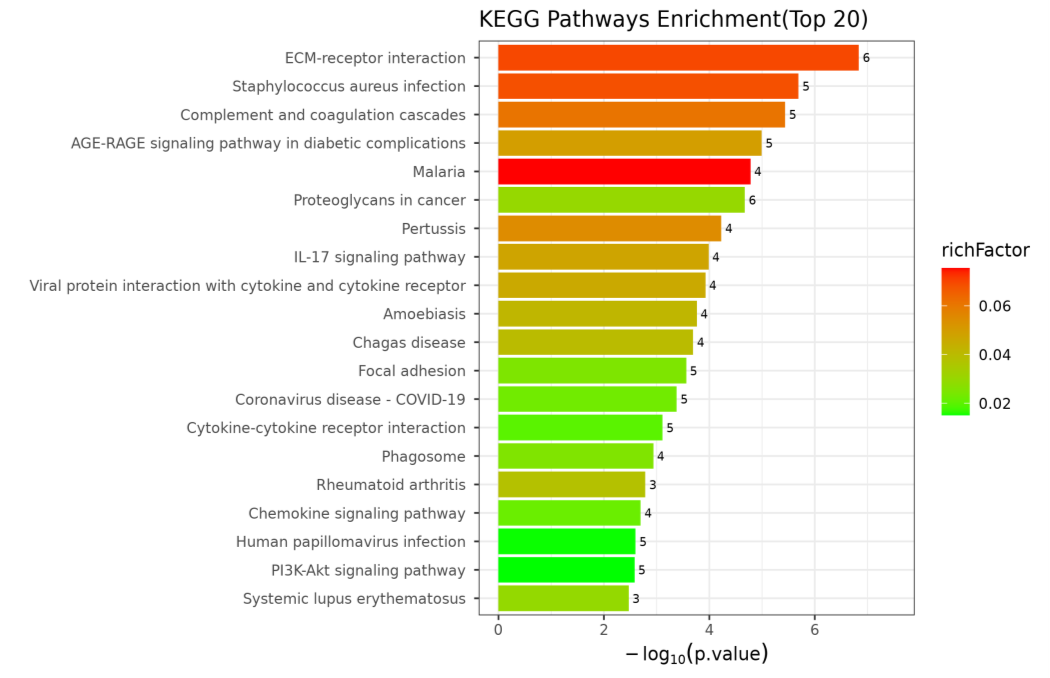


Fig S4. Bubble plot of all KEGG pathway analyses of differentially expressed genes.


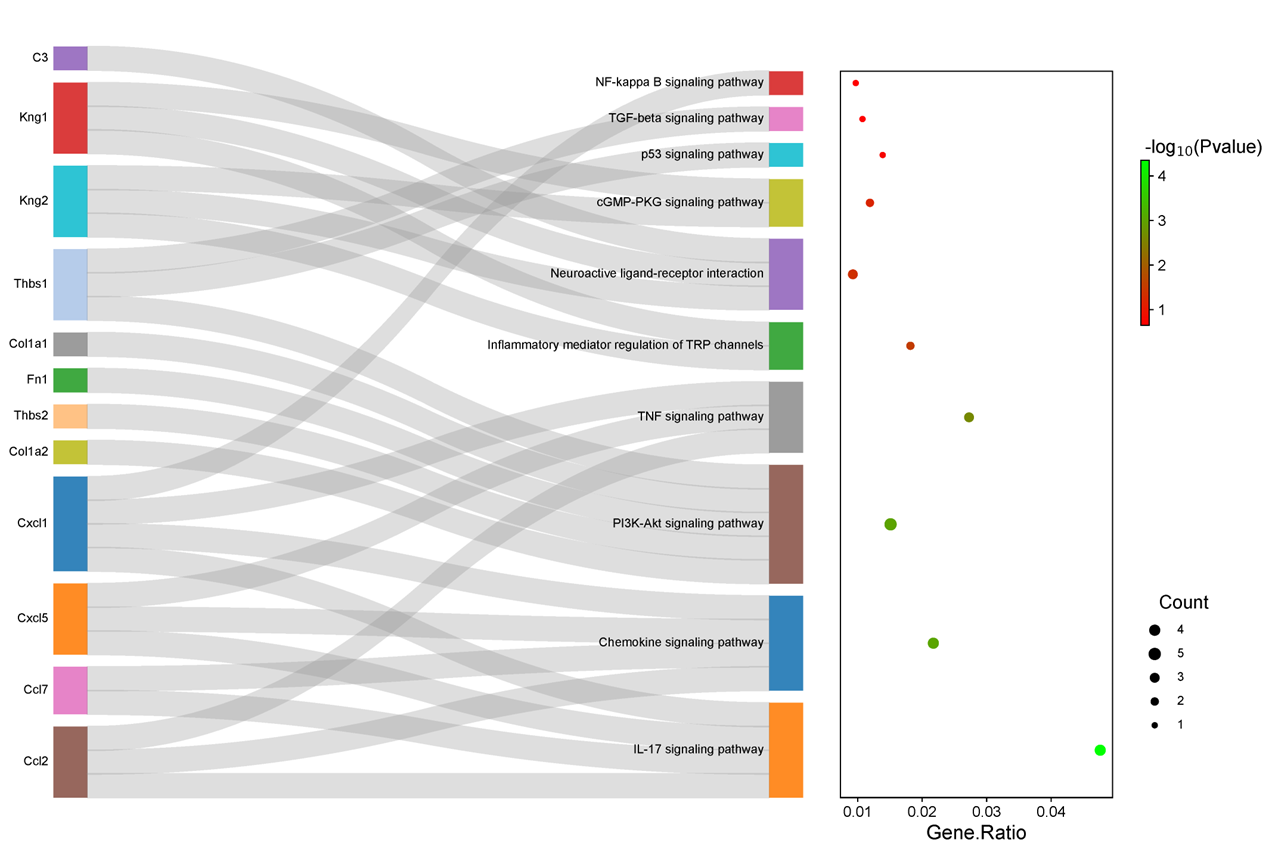


Fig S5. Molecular signaling pathways in KEGG analysis of upregulated DEG.


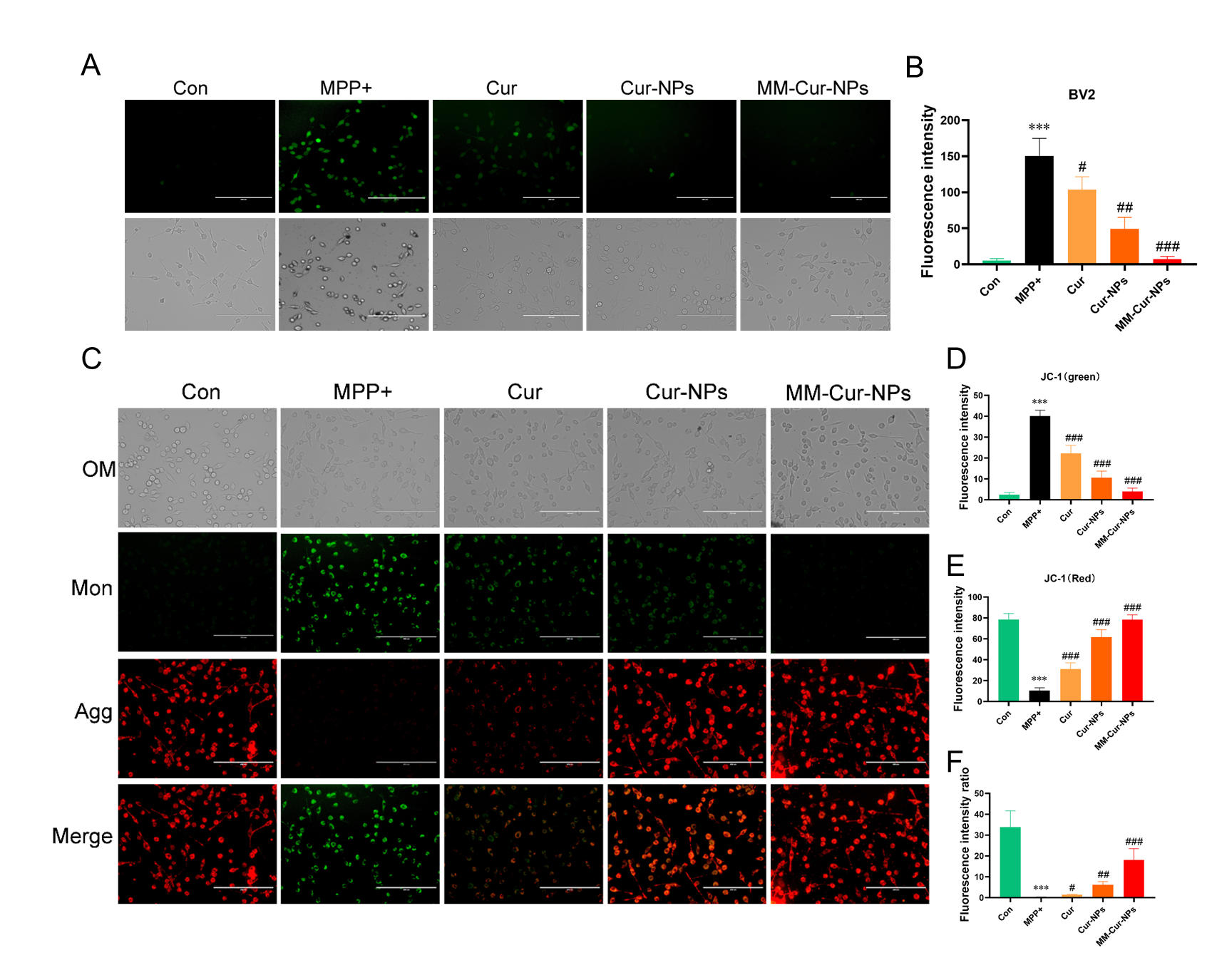


Fig S6. MM-Cur-NPs inhibits oxidative stress in microglia and repairs mitochondria. (A) ROS detection of BV2 by MPP+ treatment and different nanoparticle treatments. Scale bar: 200 μm. (B) Statistical plot of fluorescence intensity of ROS of BV2. (C) Mitochondrial staining of BV2 is observed by JC-1 staining. Scale bar: 200μm. (D) Statistical plot of green fluorescence intensity of JC-1 for BV2. (E) Statistical plot of red fluorescence intensity of JC-1 for BV2. (F) Statistical plot of the ratio of green fluorescence and red fluorescence of JC-1. The data are mean ± SD; **P* < 0.05; ***P* < 0.01; ****P* < 0.001 compared to the control group. #*P* < 0.05; # #*P* < 0.01; # # #*P* < 0.001 compared to the MPP+ group.


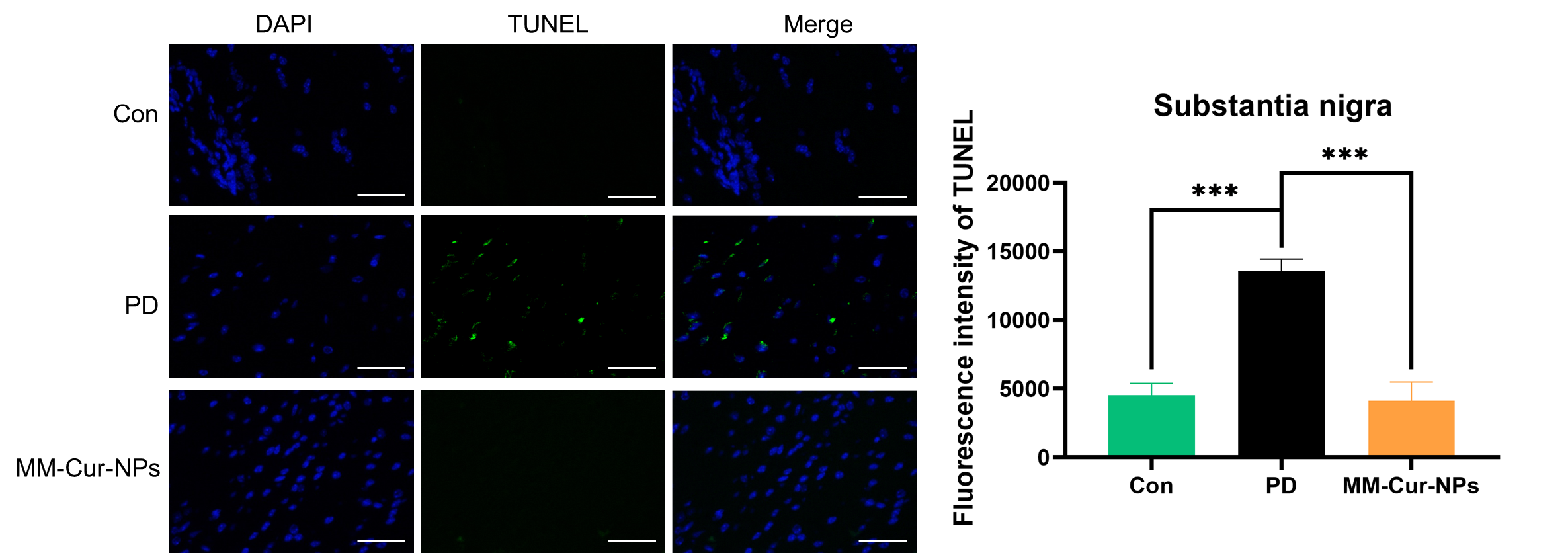


Fig S7. TUNEL of substantium nigra in PD mice treated by MM-Cur-NPs. Scale bar: 50 μm. The data are mean ± SD; **P* < 0.05; ***P* < 0.01; ****P* < 0.001.


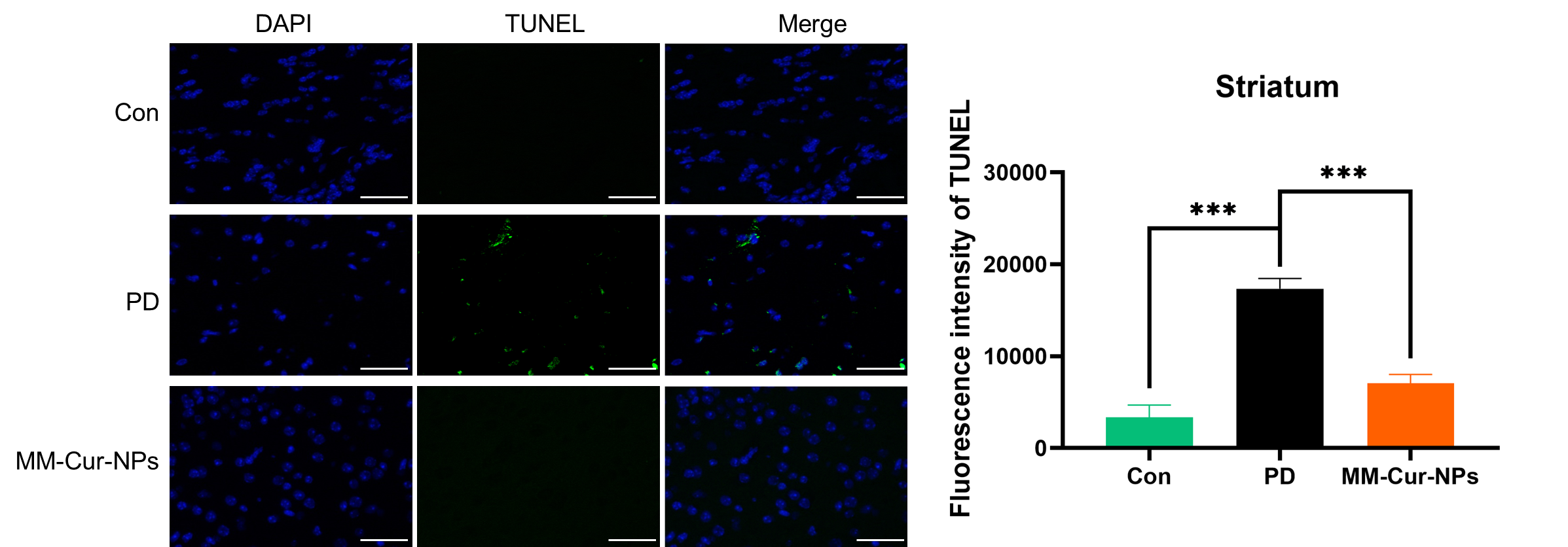


Fig S8. TUNEL of striatum in PD mice treated by MM-Cur-NPs. Scale bar: 50 μm. The data are mean ± SD; **P* < 0.05; ***P* < 0.01; ****P* < 0.001.


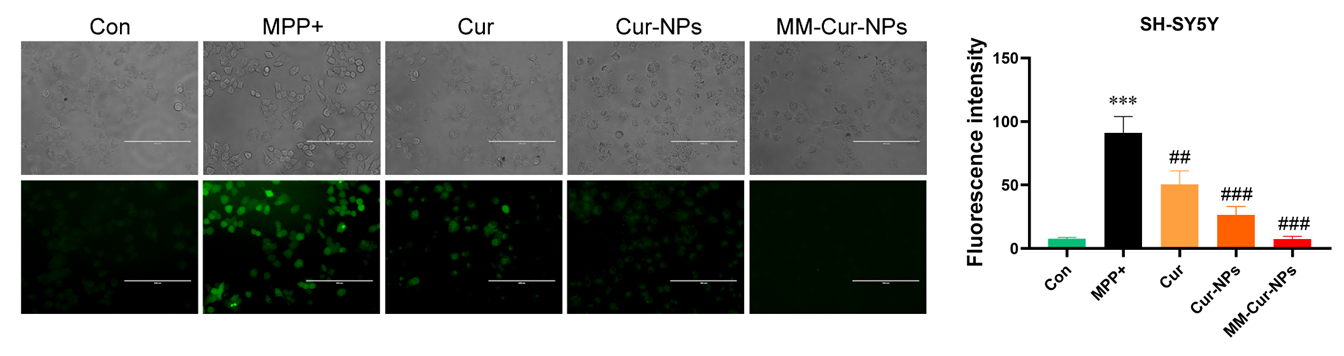


Fig S9. MM-Cur-NPs inhibits oxidative stress in SH-SY5Y. Scale bar: 200 μm. The data are mean ± SD; **P* < 0.05; ***P* < 0.01; ****P* < 0.001 compared to the control group. #*P* < 0.05; # #*P* < 0.01; # # #*P* < 0.001 compared to the MPP+ group.
